# ZNF217 confers resistance to the pro-apoptotic signals of paclitaxel and aberrant expression of Aurora-A in breast cancer cells

**DOI:** 10.1186/1476-4598-9-291

**Published:** 2010-11-08

**Authors:** Aurélie Thollet, Julie A Vendrell, Léa Payen, Sandra E Ghayad, Sabrina Ben Larbi, Evelyne Grisard, Colin Collins, Marie Villedieu, Pascale A Cohen

**Affiliations:** 1 Université de Lyon, Lyon, France; 2 ISPB, Faculté de Pharmacie de Lyon, Université Lyon 1, Lyon, France; 3 INSERM, U590, Lyon, France; 4 Centre Léon Bérard, FNCLCC, Lyon, France; 5 Departement of urology, Vancouver Prostate Centre, Vancouver, Canada

## Abstract

**Background:**

ZNF217 is a candidate oncogene located at 20q13, a chromosomal region frequently amplified in breast cancers. The precise mechanisms involved in ZNF217 pro-survival function are currently unknown, and utmost importance is given to deciphering the role of ZNF217 in cancer therapy response.

**Results:**

We provide evidence that stable overexpression of ZNF217 in MDA-MB-231 breast cancer cells conferred resistance to paclitaxel, stimulated cell proliferation *in vitro *associated with aberrant expression of several cyclins, and increased tumor growth in mouse xenograft models. Conversely, siRNA-mediated silencing of ZNF217 expression in MCF7 breast cancer cells, which possess high endogenous levels of ZNF217, led to decreased cell proliferation and increased sensitivity to paclitaxel. The paclitaxel resistance developed by ZNF217-overexpressing MDA-MB-231 cells was not mediated by the ABCB1/PgP transporter. However, ZNF217 was able to counteract the apoptotic signals mediated by paclitaxel as a consequence of alterations in the intrinsic apoptotic pathway through constitutive deregulation of the balance of Bcl-2 family proteins. Interestingly, ZNF217 expression levels were correlated with the oncogenic kinase Aurora-A expression levels, as ZNF217 overexpression led to increased expression of the Aurora-A protein, whereas ZNF217 silencing was associated with low Aurora-A expression levels. We showed that a potent Aurora-A kinase inhibitor was able to reverse paclitaxel resistance in the ZNF217-overexpressing cells.

**Conclusion:**

Altogether, these data suggest that ZNF217 might play an important role in breast neoplastic progression and chemoresistance, and that Aurora-A might be involved in ZNF217-mediated effects.

## Background

In breast cancer, the 20q13 region is amplified in up to 29% of tumors and is associated with early stage, aggressive phenotype and poor clinical prognosis [1]. A number of genes located on chromosome 20q13, such as *AURKA/STK15 *[2], *EEF1A2 *[3] and *ZNF217 *[4], appear as possible oncogenic targets of amplification. *ZNF217 *amplification correlates with shorter patient survival in breast [5] and in ovarian cancers [6]. The first direct evidence for a potentially oncogenic function of *ZNF217 *was the demonstration that the transduction of finite life-span human mammary epithelial cells with *ZNF217 *could give rise to immortalized cells with increased telomerase activity and stabilized telomere length [7]. It has been hypothesized that the selective amplification of *ZNF217 *allows cancer cells to overcome senescence and become immortal, a requirement likely essential for cancer development [8]. In support of this original study, *ZNF217 *has also recently been shown to immortalize ovarian cells [9].

ZNF217 is a Krüppel-like zinc finger protein that localizes to the nucleus [10] and interacts with co-repressors and histone modifying proteins [11-13], suggesting that ZNF217 may be part of a transcriptional repressor complex. ZNF217 promotes cell viability in HeLa cells by interfering with the apoptotic pathway and attenuates apoptotic signals resulting from doxorubicin-induced DNA damage or from functionally compromised telomeres [14]. Silencing *ZNF217 *in ovarian cells suppresses the formation of cell colonies and invasion [15]. Finally, activation of the Akt pathway [14] and overexpression of the oncogenic translation elongation factor eEF1A2 [16] have been proposed to mediate ZNF217 tumorigenic functions, but the precise molecular mechanisms involved in ZNF217 pro-survival function are currently unknown.

This study aimed to decipher the contribution of ZNF217 in cancer therapy response and to determine whether ZNF217 is able to counteract apoptotic signals other than those induced by DNA damage stimuli. Taxanes are microtubule-stabilizing agents that, by interfering with spindle microtubule dynamics, cause cell cycle arrest and apoptosis. While paclitaxel is recognized as an extremely active chemotherapeutic agent in the treatment of early-stage or metastatic breast cancers, resistance to paclitaxel has become a major concern [17]. In this study, we investigated the functional consequences of aberrant ZNF217 expression on breast cancer cell behavior. We found that ZNF217 confers a highly proliferative and paclitaxel-resistant phenotype to MDA-MB-231 breast cancer cells. To decipher the molecular mechanisms likely responsible for such phenotype, we investigated the possible involvement of the ABCB1/Pgp transporter, of the intrinsic apoptotic pathway and of the oncogenic kinase Aurora-A.

## Results

### Establishment of stable ZNF217 transfectants of breast cancer cells

**Figure 1:**
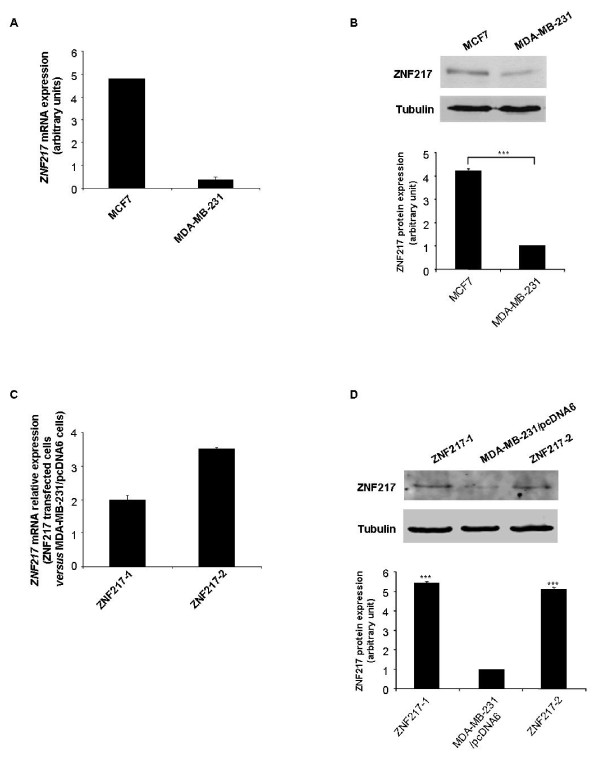
**ZNF217 expression in MCF7, MDA-MB-231 and pcDNA6/V5-His-ZNF217-transfected MDA-MB-231 cells**. (**A**) *ZNF217 *mRNA expression was analyzed by RTQ-PCR in MCF7 and MDA-MB-231 cells (means ± s.d. of three independent experiments). (**B**) western-blot analysis of ZNF217 in MCF7 and MDA-MB-231 cell lines. Histograms represent quantification of protein expression levels normalized to tubulin expression (means ± s.d. of three independent experiments). ***, *P *< 0.001 (Student's t-test). (**C**) The same as (**A**) in ZNF217-overexpressing MDA-MB-231 cells, ZNF217-1 and ZNF217-2. (**D**) The same as (**B**) in ZNF217-1, ZNF217-2 and in control MDA-MB-231/pcDNA6 cells.

With the aim of selecting relevant breast cancer cell lines to study the impact of *ZNF217 *expression on breast cancer cell phenotype, we analyzed *ZNF217 *mRNA and ZNF217 protein levels in MCF7 and MDA-MB-231 breast cancer cells. As shown in Figures 1A and 1B, MCF7 and MDA-MB-231 cells possess, respectively, high and low endogenous ZNF217 mRNA and protein levels. The high expression level of ZNF217 in MCF7 cells is consistent with the amplification of the 20q13 region in these cells [4]. However, this correlation was more difficult to establish in MDA-MB-231 cells, as the 20q13 genomic status in these cells is controversial [18,19]. Given that MDA-MB-231 cells possess low endogenous levels of ZNF217, they were used to establish stable MDA-MB-231 cells constitutively overexpressing the ZNF217 protein. After blasticidin selection, two cell clones overexpressing *ZNF217 *mRNA and ZNF217 protein (named ZNF217-1 and ZNF217-2), as well as a control cell clone transfected with the empty pcDNA6/V5-His vector (called MDA-MB-231/pcDNA6), were selected. *ZNF217 *mRNA levels were respectively 2.0- and 3.5-fold greater in ZNF217-1 and ZNF217-2 cells than in MDA-MB-231/pcDNA6 controls (Figure 1C). Accordingly, ZNF217 protein expression was increased by 5.4- and 5.1-fold in ZNF217-1 and ZNF217-2 cells, respectively, as compared to controls (Figure 1D).

### Constitutive expression of ZNF217 in MDA-MB-231 breast cancer cells promotes cell proliferation *in vitro *and tumor growth *in vivo*

**Figure 2:**
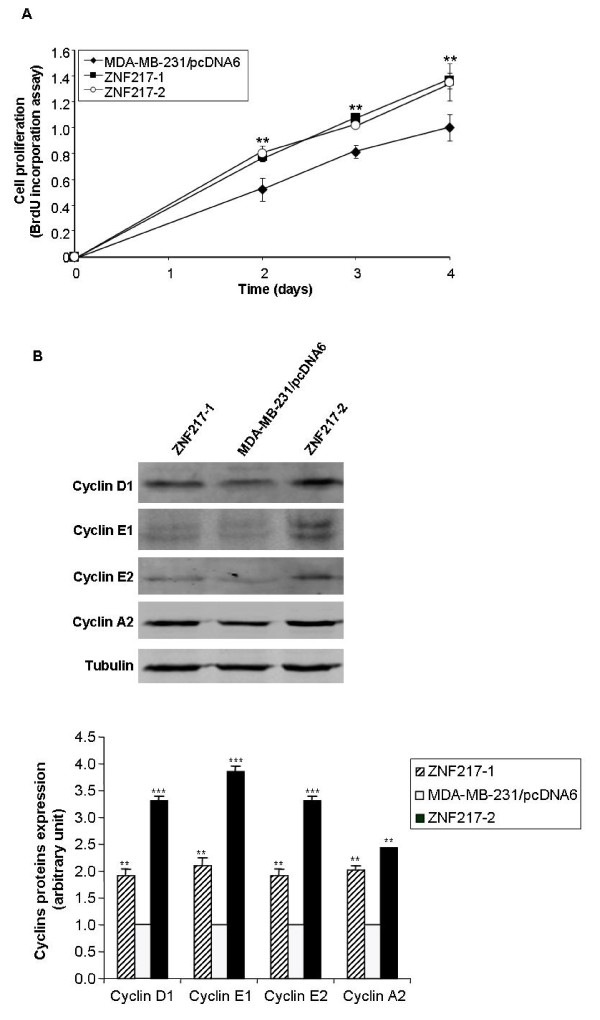
**Constitutive expression of ZNF217 stimulates cell proliferation *in vitro***. (**A**) Cell proliferation was assessed at different time points by BrdU labeling (means ± s.d. of three independent experiments). **, *P *< 0.01 *versus *MDA-MB-231/pcDNA6 cells (Student's t-test). (**B**) Western-blot analysis of Cyclin D1, Cyclin E1, Cyclin E2 and Cyclin A2. Histograms represent quantification of protein expression levels normalized to tubulin expression (means ± s.d. of three independent experiments). **, *P *< 0.01 and ***, *P *< 0.001 *versus *MDA-MB-231/pcDNA6 cells (Student's t-test).

**Figure 3:**
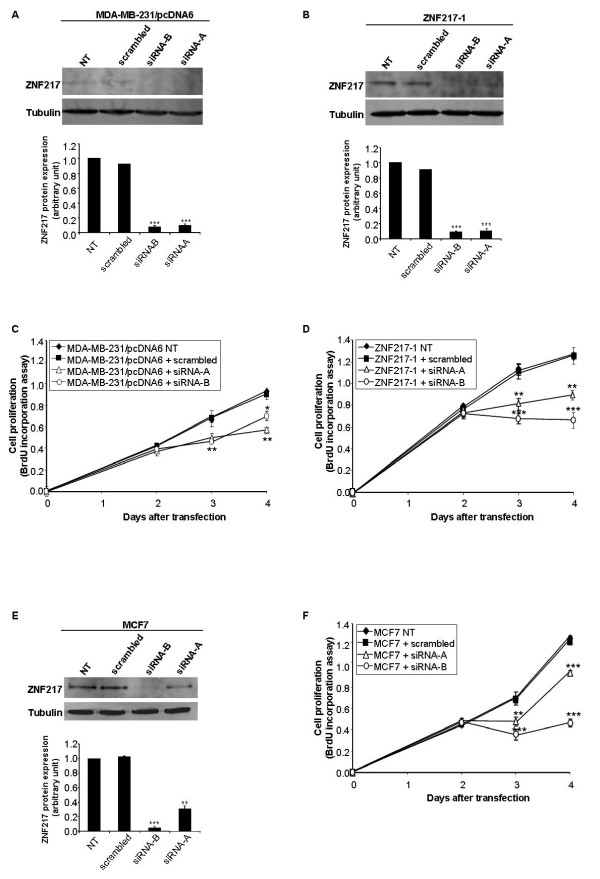
**Decreased levels of ZNF217 negatively regulates cell proliferation *in vitro***. (**A**) Western-blot analysis of ZNF217 expression in non-transfected (NT) or transfected MDA-MB-231/pcDNA6 cells with either scrambled RNA, siRNA-B or siRNA-A. Histograms represent quantification of protein expression levels normalized to tubulin expression (means ± s.d. of three independent experiments). (**B**) the same as (**A**) using the ZNF217-1 cell line. (**C**) Cell proliferation of non-transfected (NT) or transfected MDA-MB-231/pcDNA6 cells with either scrambled RNA, siRNA-B or siRNA-A as assessed by BrdU test. (**D**) the same as (**C**) using the ZNF217-1 cell line. (**E**) and (**F**) respectively the same as (**A**) and (**C**) using MCF7 cells. *, *P *< 0.05, **, *P *< 0.01 and ***, *P *< 0.001 *versus *cells transfected with scrambled RNA (Student's t-test).

**Figure 4:**
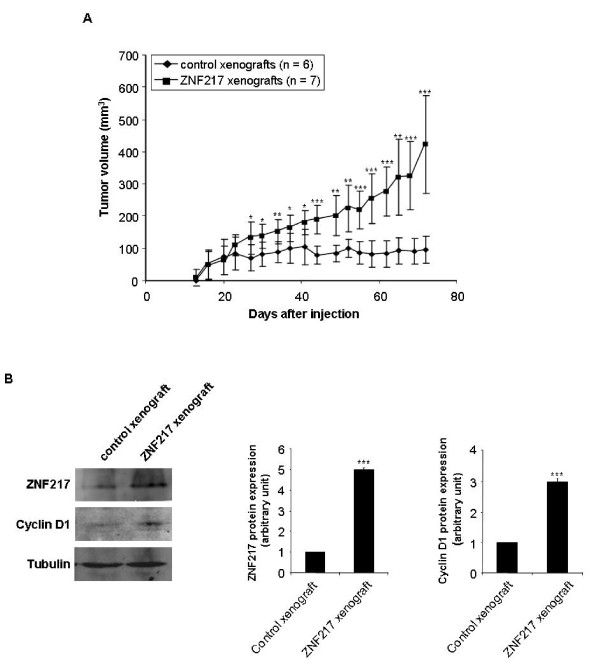
**Constitutive expression of ZNF217 stimulates tumor growth *in vivo***. (**A**) Growth curves of control xenografts (n = 6) and ZNF217 xenografts (n = 7) in nude mice. Data are presented as means ± s.d. of tumor volumes. *, *P *< 0.05, **, *P *< 0.01 and ***, *P *< 0.001 (Student's t-test). (**B**) Representative total protein extracts from control and ZNF217 xenografts were analyzed by western-blot with anti-ZNF217 and anti-Cyclin D1 antibodies. Histogram represents quantification of protein expression levels normalized to tubulin expression (means ± s.d. of three independent experiments). ***, *P *< 0.001 (Student's t-test).

By performing a BrdU incorporation assay (measurement of the proportion of cells entering S phase), we found that the constitutive expression of ZNF217 led to a significant increased proliferation of both ZNF217-1 and ZNF217-2 cells, compared to MDA-MB-231/pcDNA6 controls (Figure 2A). The ability of ZNF217 clones to proliferate more rapidly was correlated with the overexpression of Cyclin D1, Cyclin E1, Cyclin E2 and Cyclin A2 proteins, as assessed by western-blot analysis (Figure 2B). Using two siRNA molecules (-A and -B) that both specifically promote the knock-down of ZNF217 expression at the mRNA (data not shown) and protein levels in MDA-MB-231/pcDNA6 and ZNF217-1 cells (Figures 3A and 3B), we could establish that ZNF217 plays a direct role in conferring stimulation of cell proliferation. Indeed, transient transfections with the two *ZNF217-*targeted siRNAs led to a significant decrease in cell proliferation both in MDA-MB-231/pcDNA6 control cells (Figure 3C) and in ZNF217-overexpressing cells ZNF217-1 (Figure 3D). Again, when targeting the high endogenous levels of ZNF217 present in MCF7 cells, a similar cytostatic activity could be obtained with the siRNAs -A and -B (Figures 3E and 3F). Interestingly, the most potent cytostatic effect was observed in the presence of siRNA-B which was able to induce complete knock-down of ZNF217 protein expression, while siRNA-A, which promotes an intermediate knock-down of ZNF217 protein, led to an intermediate but still significant decrease in cell proliferation (Figures 3E and 3F). Taken together, these data suggest that: (i) even though breast cancer cells possess low ZNF217 levels, knock-down of ZNF217 endogenous protein expression dramatically affects their proliferation (Figures 3C and 3F); (ii) the negative regulation of cell proliferation observed with decreased levels of ZNF217 is exerted in a dose-dependent manner (Figure 3F). Finally, we examined whether the constitutive expression of ZNF217 in MDA-MB-231 cells would affect their growth in nude mice. To address this question, xenografts were established by injecting MDA-MB-231/pcDNA6 control cells or ZNF217-1 cells into the mammary fat pads of female nude mice. A significant increase in tumor growth was observed in mice injected with ZNF217-1 cells as compared with those receiving control cells (Figure 4A). Western-blot analysis of protein lysates collected from xenografts confirmed the high expression levels of both ZNF217 and Cyclin D1 in ZNF217 tumors (Figure 4B).

### ZNF217 overexpression confers paclitaxel resistance in MDA-MB-231 cells

**Figure 5:**
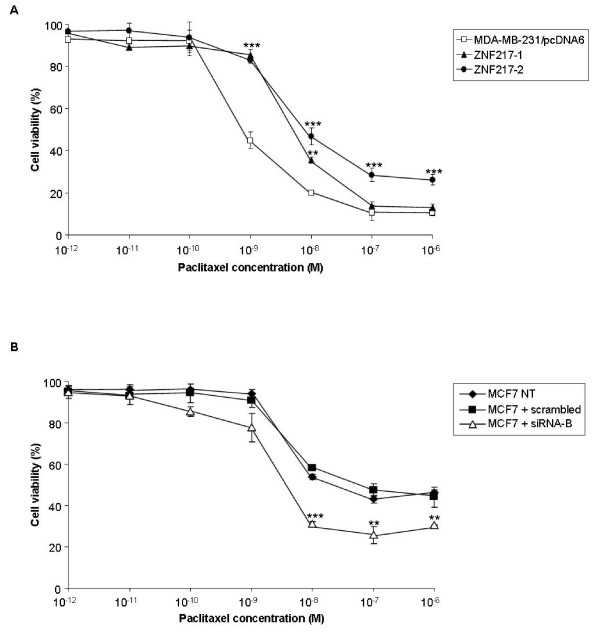
**ZNF217 expression induces resistance to paclitaxel**. Cell viability of (**A**) MDA-MB-231/pcDNA6, ZNF217-1 and ZNF217-2 cells and (**B**) non-transfected (NT) or transfected MCF7 cells with either scrambled RNA or siRNA-B was assessed by cytotoxicity assay (means ± s.d. from three independent experiments). **, *P *< 0.01, ***, *P *< 0.001 (Student's t-test).

To test whether ZNF217 alters response to chemotherapy, we performed dose-response experiments (cytotoxicity assay) to measure IC50 values under two cytotoxic stimuli: paclitaxel and gemcitabine. Strikingly, constitutive expression of ZNF217 led to significant increased cell viability in the presence of the microtubule-stabilizing agent paclitaxel (Figure 5A), with a relative resistance of 7.5- and 12-fold, respectively, for ZNF217-1 and ZNF217-2 cells (IC50_MDA-MB-231/pcDNA6 _= 6.5 ± 1.4×10^-10 ^M, IC50_ZNF217-1 _= 4.9 ± 1.7×10^-9 ^M, IC50_ZNF217-2 _= 7.8 ± 0.6×10^-9 ^M). Interestingly, MCF7 cells, which possess high endogenous levels of ZNF217, displayed lower sensitivity to paclitaxel (IC50_MCF7 _= 2.2 ± 0.7×10^-7 ^M) than MDA-MB-231/pcDNA6 cells (Figure 5B). Moreover, the knock-down of ZNF217 expression in MCF7 cells by transient transfections with siRNA-B conferred increased sensitivity to paclitaxel (IC50_siRNA-B-transfected MCF7 _= 4 ± 0.7×10^-9 ^M, IC50_scrambled-transfected MCF7 _= 2.2 ± 0.7×10^-7 ^M) (Figure 5B). These data suggest that ZNF217 is able to modulate the cellular response to paclitaxel and that the constitutive expression of ZNF217 supports the survival of MDA-MB-231 cells in response to this microtubule-stabilizing molecule. In contrast, no significant difference in gemcitabine response could be observed between ZNF217-overexpressing cells and controls (data not shown). Finally, paclitaxel resistance could also be observed in an additional breast cancer cell line (MDA-MB-453) stably transfected with ZNF217 when compared to control cells (data not shown), suggesting that the paclitaxel-resistant phenotype developed by ZNF217-overexpressing cells occurs in different breast cancer cell lines.

### ABCB1/PgP transporter is not involved in the paclitaxel resistance developed by ZNF217-overexpressing cells

**Figure 6:**
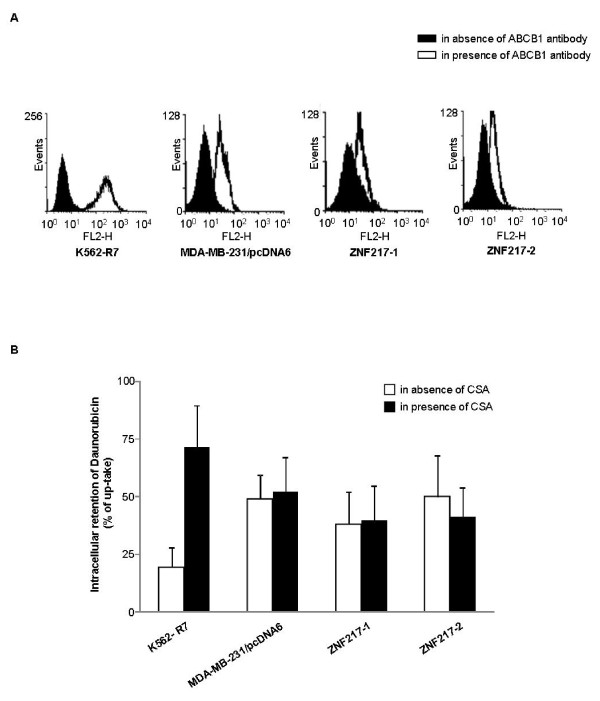
**ABCB1/PgP transporter does not mediate ZNF217-induced resistance to paclitaxel**. (**A**) ABCB1 protein levels were analyzed by flow cytometry in control K562-R7, MDA-MB-231/pcDNA6, ZNF217-1 and ZNF217-2 cells. Representative FACS histograms of the three cell lines after incubation with or without ABCB1 antibody were superimposed according to increased PE fluorescence. (**B**) Intracellular DNR efflux was assessed by flow cytometry. Maximal DNR accumulation (100%) is represented by the DNR fluorescence median after 30 min accumulation. After DNR removal, cells were incubated 1 h in the absence (white columns) or in the presence of CSA (black columns). Results are means ± s.d. from three independent experiments.

Cancer cells frequently exhibit multidrug resistance mediated by ATP-binding cassette (ABC) membrane proteins. The ABCB1/PgP protein efficiently transports taxanes and its overexpression induces resistance to paclitaxel [20]. We thus evaluated ABCB1 expression levels and transport capabilities in MDA-MB-231/pcDNA6 controls, ZNF217-1 and ZNF217-2 cells. Very low and similar ABCB1 endogenous expression levels were detected in all three cell lines, while it was strongly detected in K562-R7 ABCB1-positive control cells (Figure 6A). To evaluate ABCB1 transport capabilities, we then used a standard test that investigates the efflux of daunorubicin (DNR), a well-known fluorescent substrate of ABCB1 that binds to the same binding sites as paclitaxel on the transporter [21-24]. Inhibitors such as cyclosporin A (CSA), a reference ABC transporter inhibitor, reduce DNR efflux in cells that have an active mechanism for the outward transport of the drug [24]. We confirmed that CSA was able to strongly block DNR efflux in K562-R7 ABCB1-positive cells (Figure 6B). DNR intracellular levels were similarly decreased in MDA-MB-231/pcDNA6, ZNF217-1 and ZNF217-2 cells after a 1 h efflux period, thus revealing no difference between the three cell lines. Moreover, as DNR intracellular levels were not altered in the presence of CSA, CSA-insensitive elimination processes were probably responsible for the DNR efflux observed in the three cell lines. Since both ABCB1 was weakly expressed in cells and DNR efflux was not modified by CSA, our data strongly suggest that ABCB1 was not involved in the paclitaxel-resistance mechanisms developed by ZNF217-overexpressing MDA-MB-231 cells.

### Paclitaxel-induced apoptotic activity is altered in ZNF217-overexpressing MDA-MB-231 cells

**Figure 7:**
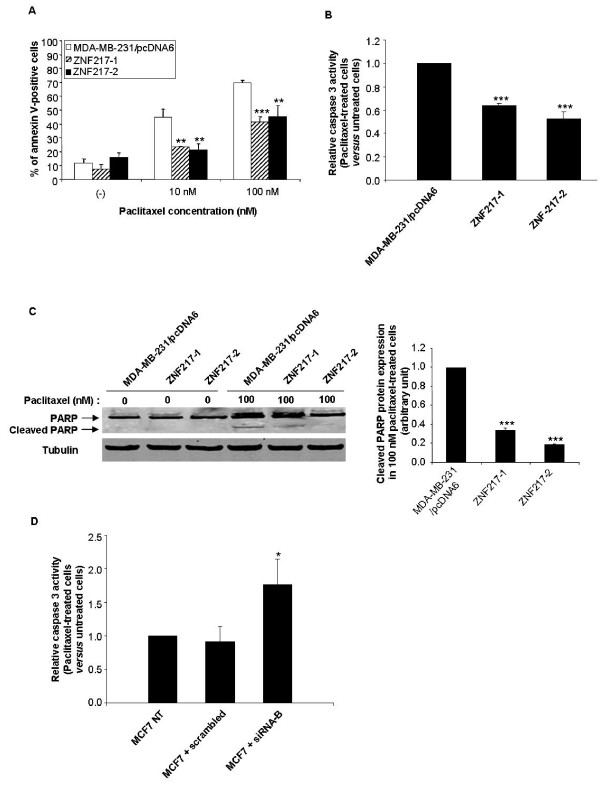
**ZNF217 overexpression alters paclitaxel-induced apoptosis**. (**A**) MDA-MB-231/pcDNA6, ZNF217-1 and ZNF217-2 cells were untreated (-) or treated with 10 or 100 nM paclitaxel, stained with Annexin-V-Fluos and propidium iodide and analyzed by flow cytometry (means ± s.d. from three independent experiments). **, *P *< 0.01 and ***, *P *< 0.001 *versus *the corresponding MDA-MB-231/pcDNA6 cells (Student's t-test). (**B**) MDA-MB-231/pcDNA6, ZNF217-1 and ZNF217-2 cells were treated with 100 nM paclitaxel. Caspase 3 activity was assessed (means ± s.d. from three independent experiments). ***, *P < 0.001 *versus MDA-MB-231/pcDNA6 cells (Student's t-test). (**C**) Western-blot analysis of PARP cleavage in response to 100 nM paclitaxel. Histogram represents quantification of the cleaved PARP in 100 nM paclitaxel-treated cells and tubulin expression was used for normalization (means ± s.d. of three independent experiments). ***, *P *< 0.001 *versus *paclitaxel-treated MDA-MB-231/pcDNA6 cells (Student's t-test). (**D**) Non-transfected (NT) or transfected MCF7 cells with either scrambled RNA or siRNA-B were treated with 100 nM paclitaxel. Caspase 3 activity was assessed (means ± s.d. from three independent experiments). *, *P *< 0.05 *versus *MCF7 cells transfected with scrambled RNA (Student's t-test).

As paclitaxel has been shown to induce apoptosis in several cell lines in a dose-dependent manner [25,26], we investigated the impact of ZNF217 expression on paclitaxel pro-apoptotic signals. We confirmed that paclitaxel was able to elicit cell death in MDA-MB-231/pcDNA6 cells in a dose-dependent manner (10 nM and 100 nM paclitaxel induced apoptosis in respectively 45.1% and 69.6% of the cells, Figure 7A). In ZNF217-overexpressing cells, no decreased spontaneous cell death could be observed at basal level compared to control cells (Figure 7A), in contrast to results reported by Huang and collaborators in ZNF217-overexpressing HeLa cells [14] and no significant difference could be observed between the three cell lines (Figure 7A). However, 10 nM and 100 nM paclitaxel strikingly elicited a significant lower apoptotic response in ZNF217-overexpressing cells than in controls (Figure 7A). As the maximum paclitaxel-induced apoptotic response was observed with 100 nM paclitaxel, this dose was chosen for further investigations. Measurement of caspase 3 activity also provided evidence that apoptotic pathways were significantly less activated in ZNF217-1 and ZNF217-2 cells than in control cells (Figure 7B). Supporting data showed that, in MDA-MB-231/pcDNA6 controls, 100 nM paclitaxel induced cleavage of the PARP protein which was only faintly detectable in ZNF217-1 cells and absent in ZNF217-2 cells (Figure 7C). Finally, transient transfection of MCF7 cells with a *ZNF217-*targeted siRNA (compared to scrambled control RNA) led to a significant increase in caspase 3 activity on paclitaxel treatment (Figure 7D), suggesting that the knock-down of ZNF217 expression in MCF7 cells confers increased sensitivity to paclitaxel pro-apoptotic signals. Altogether, these data suggest that constitutive ZNF217 expression confers resistance to paclitaxel-mediated apoptosis.

### Acquired resistance to paclitaxel in ZNF217-overexpressing cells is mediated by alterations of proteins of the Bcl-2 family implicated in the mitochondrial apoptosis pathway

**Figure 8:**
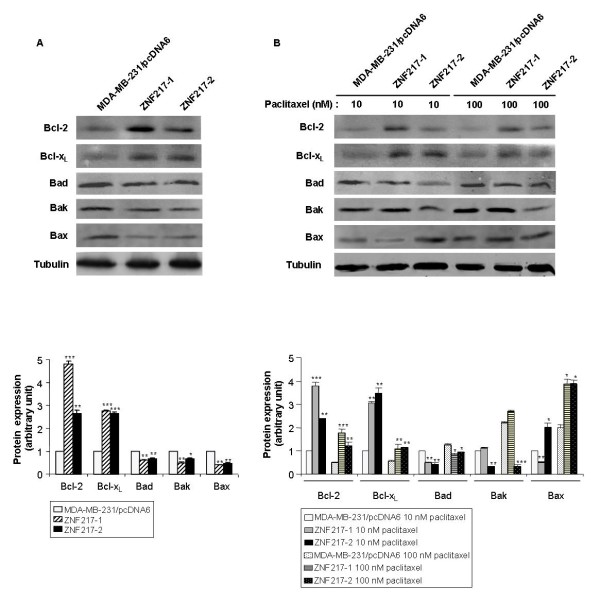
**ZNF217 overexpression is associated with altered expression of Bcl-2 family proteins**. (**A**) Western-blot analysis of Bcl-2, Bcl-x_L_, Bad, Bak, Bax at basal level or (**B**) after 3 days of paclitaxel treatment. Histograms represent quantification of protein expression levels normalized to tubulin expression (means ± s.d. of three independent experiments). *, *P *< 0.05, **, *P *< 0.01 and ***, *P *< 0.001 *versus *the corresponding MDA-MB-231/pcDNA6 cells (Student's t-test).

In breast cancer cells that acquired resistance to paclitaxel, it has been recently demonstrated that the mitochondrial (intrinsic) apoptosis pathway controlled by Bcl-2 protein family members is crucial for causing such resistance [27]. We thus examined whether changes in the mitochondrial apoptotic pathway were selected for in ZNF217-overexpressing cells. Because we were interested by permanent changes established under constitutive expression of ZNF217, we first evaluated the effect of ZNF217 on the levels of several Bcl-2 family proteins. In the two ZNF217-overexpressing cell lines studied, we observed a constitutive overexpression of the anti-apoptotic proteins Bcl-2 and Bcl-x_L _and an under-expression of the pro-apoptotic proteins Bad, Bak and Bax (Figure 8A), as compared to MDA-MB-231/pcDNA6 controls. Moreover, as shown in Figure 8B, treatment with 10 nM or 100 nM paclitaxel was still associated with the overexpression of Bcl-2 and Bcl-x_L _and the down-regulation of Bad in both ZNF217-1 and ZNF217-2 cell lines. The persistence of the deregulated expression levels observed for Bcl-2, Bcl-x_L _and Bad under paclitaxel exposure was less obvious for Bak and Bax proteins, as it depended on the paclitaxel dose and on the ZNF217-overexpressing clone considered. Taken together, these results indicate that the resistance to paclitaxel displayed by ZNF217-overexpressing cells might be promoted by deregulations of the intrinsic apoptosis pathway through aberrant expression of several members of the Bcl-2 family.

### Increased protein expression of the Aurora kinase A/AURKA/STK15 is correlated with constitutive expression of ZNF217 and ZNF217-mediated paclitaxel resistance is reversed in the presence of an Aurora-A inhibitor

**Figure 9:**
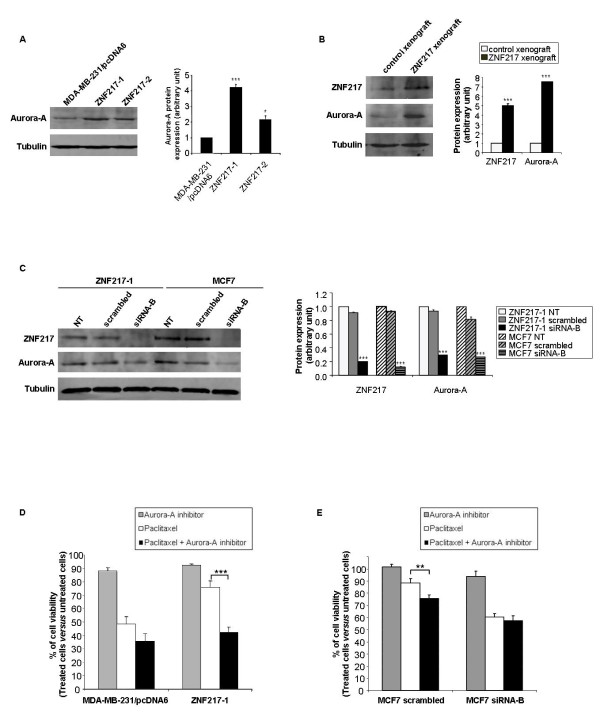
**ZNF217 modulates Aurora-A protein expression and ZNF217-mediated paclitaxel resistance is reversed by an Aurora-A inhibitor**. Western-blot analysis of Aurora-A expression in (**A**) MDA-MB-231/pcDNA6, ZNF217-1 and ZNF217-2 cells, (**B**) in representative control and ZNF217 xenograft cells, (**C**) in ZNF217-1 or MCF7 cells transfected or not (NT) with either scrambled RNA or siRNA-B. (**A**), (**B**) and (**C**) Histograms represent quantification of protein expression levels normalized to tubulin expression (means ± s.d. of three independent experiments). *, *P *< 0.05 and ***, *P *< 0.001 (Student's t-test). (**D**) The viability of MDA-MB-231/pcDNA6 and ZNF217-1 cells treated with 5 μM Aurora-A kinase inhibitor III, 2.5 nM paclitaxel or a combination of both was assessed by cytotoxicity assay (means ± s.d. from three independent experiments). ***, *P *< 0.001 (Student's t-test). (**E**) MCF7 cells transfected with either scrambled RNA or siRNA-B were treated with 1 μM Aurora-A kinase inhibitor III, 2.5 nM paclitaxel or a combination of both. Cell viability was assessed by cytotoxicity assay (means ± s.d. from three independent experiments). **, *P *< 0.01 (Student's t-test).

Since high expression levels of Aurora-A have been associated with increased taxane resistance in breast cancer and with resistance to taxol-mediated apoptosis in breast cancer cell lines [28,29], we investigated Aurora-A expression levels in our cellular models. Strikingly, high protein expression levels of Aurora-A could be detected in the two ZNF217-overexpressing cell lines (Figure 9A) and in ZNF217 xenografts (Figure 9B). ZNF217 was demonstrated to play a direct role in Aurora-A overexpression, as transient transfections with a *ZNF217-*targeted siRNA led to a significant decrease in Aurora-A protein expression both in ZNF217-1 and in MCF7 cells (that possess naturally high endogenous levels of ZNF217) (Figure 9C). As ZNF217 has been identified as a transcription factor, we explored by RTQ-PCR *AURKA *transcript levels in MDA-MB-231/pcDNA6, ZNF217-1, and ZNF217-2 cells, but we did not find any difference between the three cell lines (data not shown). This suggests that the ZNF217-mediated aberrant expression of Aurora-A is probably controlled by a post-transcriptional mechanism.

With the aim to decipher whether Aurora-A is involved in the paclitaxel resistance developed by the ZNF217-overexpressing cells, we then sought to investigate the impact of an Aurora-A kinase inhibitor on paclitaxel response. The Aurora kinase inhibitor III is a compound known to act as an ATP-competitive and potent inhibitor of Aurora-A (Merck). This inhibitor (5 μM) exerted a weak and similar inhibitory effect (~10%) on viability of both MDA-MB-231/pcDNA6 and ZNF217-1 cells (Figure 9D). In control MDA-MB-231/pcDNA6 cells, combining 5 μM Aurora-A kinase inhibitor with paclitaxel led to an additive inhibitory effect of the two molecules on cell viability (51.5% inhibition under paclitaxel exposure and 63.6% inhibition when combining the Aurora-A kinase inhibitor with paclitaxel). Strikingly, in ZNF217-1 cells, combining 5 μM Aurora-A kinase inhibitor with paclitaxel potentialized paclitaxel cytotoxic effect (Figure 9D, 57% of inhibition of cell viability in Aurora-A kinase inhibitor- and paclitaxel-treated ZNF217-1 cells compared to 24% inhibition of cell viability in paclitaxel-treated ZNF217-1 cells, *P *< 0.001), and induced a pharmacological response close to that of control paclitaxel-treated MDA-MB-231/pcDNA6 cells. We next investigated the impact of the Aurora-A kinase inhibitor on paclitaxel response in MCF7 cells transfected with either a *ZNF217-*targeted siRNA or scrambled RNA (Figure 9E). In MCF7 cells transiently transfected with scrambled control RNA (*i.e*. still possessing high endogenous levels of ZNF217), combining the Aurora-A kinase inhibitor with paclitaxel led to an additive inhibitory effect on cell viability, as observed in ZNF217-overexpressing MDA-MB-231 cells (Figure 9D). More interestingly, combining paclitaxel with the Aurora-A inhibitor in MCF7 cells transiently transfected with a *ZNF217-*targeted siRNA induced no significant alteration of cell viability (as observed in the control MDA-MB-231/pcDNA6 cells that possess low endogenous levels of ZNF217, Figure 9D). Taken together, these data strongly suggest that the impact of the Aurora-A inhibitor on paclitaxel response is clearly associated with ZNF217 expression levels. Therefore Aurora-A is certainly part of the mechanism by which ZNF217 controls resistance to paclitaxel, and the use of a potent Aurora-A inhibitor could be sufficient to reverse ZNF217-mediated paclitaxel resistance in MDA-MB-231 breast cancer cells.

## Discussion

It has been shown that ZNF217 is able to immortalize human mammary epithelial cells, to overcome senescence and to attenuate apoptotic signals emanating from DNA damage after doxorubicin exposure or from functionally compromised telomeres [7,14]. The precise mechanisms involved in ZNF217 pro-survival function are currently unknown, and it is thus of utmost importance to decipher the role of ZNF217 in response to cancer therapy.

In this study, we provide evidence that ectopic expression of ZNF217 in MDA-MB-231 breast cancer cells is associated with a highly proliferative phenotype, as constitutive expression of ZNF217 stimulates breast cancer cell proliferation *in vitro *and tumor growth *in vivo *in association with aberrant expression of several cyclins. We have also found that high expression levels of ZNF217 in MDA-MB-231 breast cancer cells promote strong resistance (~10-fold) to the microtubule-stabilizing molecule paclitaxel, while no resistance to the nucleoside analogue gemcitabine is concomitantly developed. In accordance with the previous observation that overexpression of ZNF217 decreases doxorubicin-induced cell death in cervical (HeLa) and breast (HBL100) cancer cell lines [14], the two ZNF217-overexpressing MDA-MB-231 cell lines studied also displayed a ~2.3-fold relative resistance to the topoisomerase inhibitor doxorubicin (data not shown). Our data suggest, firstly, that the chemoresistance mediated by ZNF217 is drug-specific and, secondly, that the molecular mechanisms involved in paclitaxel response are probably more sensitive to the ZNF217-mediated protective action than those involved in response to doxorubicin.

After pointing out that the ABCB1 transporter, known to be involved in taxane and doxorubicin transport, is not responsible for the resistant phenotype developed by ZNF217-overexpressing cells, we aimed to determine which molecular mechanisms are involved in the ZNF217-mediated phenotype. A critical determinant in cellular responses to cytotoxic drugs is the ease with which tumor cells undergo apoptosis [30]. The two major apoptotic pathways rely on either signals transduced through death receptors or signals from mitochondria. Both pathways are involved in the activation of a cascade of caspases, and caspase 3 is a major executioner caspase that cleaves substrates such as PARP, resulting in caspase-dependent apoptosis [31]. In this study, we found that ZNF217 attenuates the apoptotic signals induced by paclitaxel, in association with decreased caspase 3 activity and PARP cleavage. More interestingly, ZNF217-mediated protective effects were associated with alterations in the intrinsic mitochondrial apoptosis pathway, as demonstrated by the deregulation of the balance between pro- and anti-apoptotic members of the Bcl-2 family. Indeed, in both ZNF217-1 and ZNF217-2 cells, high constitutive expression levels of the anti-apoptotic proteins Bcl-2 and Bcl-x_L _and low constitutive expression levels of the pro-apoptotic proteins Bad, Bax and Bak were detected. Since ectopic overexpression of Bcl-2 or Bcl-x_L _is necessary to confer resistance to paclitaxel-induced apoptosis [32] and ectopic overexpression of Bad or Bax have been shown to enhance paclitaxel-induced apoptosis [33], our data strongly suggest that acquisition of aberrant expression of several members of the Bcl-2 family may be part of the mechanisms developed by ZNF217-1 and ZNF217-2 cells to counteract paclitaxel-induced apoptotic signals.

The p53 pathway has been suggested to be involved in ZNF217 functions [14], but the ZNF217-driven survival phenotype observed in this study is p53-independent, given that MDA-MB-231 cells possess a non functional mutated p53.

Aurora-A, a serine/threonine kinase located at the centrosome, is overexpressed in 10-60% of breast cancers [29], functions as a pro-survival protein that promotes tumor cell proliferation, counteracts apoptosis and induces drug resistance in tumor cells [29,34]. Indeed, Aurora-A overexpression has been associated in cancer cells with spindle checkpoint dysfunction, increased resistance to paclitaxel and docetaxel [28,29], increased expression of Bcl-2 [34] or of Bcl-x_L _[35]. Conversely, Aurora kinase inhibitors synergize with paclitaxel to induce apoptosis in ovarian cancer cells [36]. In this study, we show for the first time that ZNF217 modulates Aurora-A expression, probably at a post-transcriptional level. Post-transcriptional mechanisms such as phosphorylation-dephosphorylation events [37] or ubiquitin-dependent proteolysis [38,39] have been shown to regulate the protein levels of Aurora-A and to play an important role in the functions of this protein [40-42]. Thus ZNF217 may modulate Aurora-A protein turn-over (synthesis or degradation) by still unknown mechanisms. We also newly demonstrated that treatment with a potent Aurora-A kinase inhibitor is able to reverse paclitaxel resistance in ZNF217-overexpressing breast cancer cells. Altogether, these data strongly suggest that the oncogenic Aurora-A kinase could represent a key actor of ZNF217-mediated effects. Finally, as observed for most of kinase inhibitors, the Aurora kinase inhibitor III is also able to target (but at higher concentrations) the activities of other kinases (Lck, Bmx, IGF-1R and Syk). Thus, one cannot exclude that these kinases could also be involved in ZNF217-mediating effects.

Since the *AURKA *gene is located, like the *ZNF217 *gene, at 20q13, a region frequently amplified in human cancers, our finding is of particular interest for several reasons. Our data suggest that two known oncogenes, ZNF217 and Aurora-A, may cooperate in breast neoplastic progression and chemoresistance, thus revealing a possible mechanism by which ZNF217 exerts its oncogenic and protective effects. The positive control of ZNF217 on Aurora-A could induce a self-reinforcing amplification of the effect of high levels of ZNF217 expression and/or of increased *ZNF217 *copy number. As 20q13 amplified breast tumors can either display *ZNF217 *amplification only or both *ZNF217 *and *AURKA *amplifications [5], one of the mechanisms of breast neoplastic progression could involve the cooperation between the two proteins, either through genomic co-amplification or through ZNF217-mediated regulation of Aurora-A protein expression.

## Conclusions

This study demonstrates that ZNF217 counteracts apoptotic signals other than those induced by DNA damage stimuli [14], and that the protective effects of ZNF217 are associated with constitutive alterations in the balance of Bcl-2 proteins and with constitutive aberrant overexpression of Aurora-A. Given that ZNF217 amplifications have been detected in 8-29% of breast cancers [1] and that high ZNF217 expression levels are not necessarily correlated to increased *ZNF217 *gene copy numbers in breast cancer cells [4,5,10], increased protein expression of ZNF217 could represent a new mechanism by which breast cancer cells without *ZNF217 *gene amplification become resistant to paclitaxel. Most importantly, our data suggest that clinical strategies counteracting ZNF217-mediated effects, either by targeting ZNF217 directly and/or by targeting its possible key-mediators like Aurora-A, would be a valuable approach for the management of breast cancer.

## Materials and methods

### Cell culture

MCF7 and MDA-MB-231 breast cancer cells were purchased from ATCC and grown according to recommendations in DMEM medium supplemented with 10% fetal bovine serum (Invitrogen, Cergy Pontoise, Paris). The identity of MDA-MB-231 cells was confirmed by genomic DNA sequencing *(KRAS *and *TP53 *genes) and that of MCF7 cells by their estrogen receptor and progesterone receptor status.

### MDA-MB-231-ZNF217 stable transfectants

The full-length *ZNF217 *cDNA was obtained by adding the missing cDNA sequence, corresponding to the last 6 C-terminal amino acids of the ZNF217 protein, to the pEGFP-N1-ZNF217 plasmid provided by C. Collins, then subcloned into the pcDNA6/V5-His plasmid (Invitrogen) (pcDNA6/V5-His-ZNF217). MDA-MB-231 breast cancer cells were stably transfected with pcDNA6/V5-His or pcDNA6/V5-His-ZNF217 plasmids, then selected in the presence of 20 μg/ml blasticidin (Invitrogen).

### Real-time quantitative PCR (RTQ-PCR)

Total RNA from cell culture was prepared using the RNeasy Mini Kit (Qiagen, Hilden, Germany). One microgram of total RNA was reversed-transcribed, and RTQ-PCR measurements were performed as described previously [43].

### Western-blot

Western-blot analysis was performed as previously described [43]. For each sample, total proteins were quantified using a Bradford protein assay and 50 μg of total protein were separated on SDS/PAGE gels before transferring to a PVDF membrane (Sigma-Aldrich, St Quentin Fallavier, France). ZNF217 antibody was obtained from C. Collins [14], Cyclin D1, PARP, Bax and Aurora-A antibodies were from Cell Signaling (Beverly, MA, USA), Cyclin E2 and Bcl-x_L _antibodies from Santa Cruz Biotechnology Inc. (Santa Cruz, CA, USA), Bcl-2 antibody from Neomarker (Fremont, CA, USA), Bad antibody from BD Biosciences (Franklin Lakes, NJ, USA), Bak and Cyclin E1 antibodies from Calbiochem (San Diego, CA, USA) and Cyclin A2 and α-tubulin antibodies from Sigma Chemical Co. (St. Louis, MO, USA). All western-blots presented are from one experiment representative of at least two independent experiments and cell lysates, and at least three western-blots. Signals were quantified by pixel densitometry using the VisionWorksLS Analysis Software.

### Gene silencing

Stealth™ siRNAs (siRNA-A and siRNA-B) targeting *ZNF217 *and scrambled control RNA (scrambled) were obtained from Invitrogen. Five nanomoles of ZNF217-siRNAs or scrambled were transfected into cell lines with lipofectamine RNAimax (Invitrogen).

### Cell proliferation analysis

Cells (4 000 cells per well) were plated onto a 96-well plate. Proliferating cells were analyzed using a Cell Proliferation ELISA 5-bromodeoxyuridine (BrdU) Kit (Roche, Meylan, France) as previously described [44].

### Tumor growth assay

A total of 2×10^6 ^MDA-MB-231/pcDNA6 or ZNF217-1 cells were suspended in PBS/matrigel v/v (BD Biosciences) and injected into the mammary fat pad of 4-week-old female Swiss nude (nu/nu) mice (Charles River, L'arbresle, France) (control xenografts n = 6 and ZNF217 xenografts n = 7). Tumors were measured with calipers every 3-4 days. All animal studies were performed in accordance with the European Union guidelines and use committee of Centre Léon Bérard.

### Cytotoxicity assay

Cells (8 000 cells per well) were plated onto a 96-well plate, treated for 4 days with 10^-12 ^to 10^-6 ^M of paclitaxel (Paxene^®^, Ivax, Miami, USA). Cell viability was then assessed with the CellTiter 96 AQueous One Solution Cell Proliferation assay (Promega, Madison, WI, USA). Cytotoxic experiments were also conducted as described above in the presence of Aurora kinase inhibitor III, a potent inhibitor of Aurora-A (Merck, Nottingham, UK), alone or combined with 2.5 nM paclitaxel.

### Flow cytometry analysis of ABCB1 protein expression levels

ABCB1 protein levels were quantified by flow cytometry in ABCB1-positive control K562-R7, MDA-MB-231/pcDNA6, ZNF217-1 and ZNF217-2 cells using the ABCB1-C219 (phycoerythrin PE)-conjugated antibody (Santa Cruz) according to the manufacturer's instructions.

### Efflux assay

At the end of uptake phase (30 min, 37°C), daunorubicin (17 μM DNR, DaunoXome^®^, San Dimas, CA, USA) was removed and cells were re-incubated for 1 h in DNR-free medium in the presence or absence of 4 μg/ml cyclosporin A (CSA). After trypsination, DNR fluorescence was monitored with a FACscan flow cytometer (Becton Dickinson, Mountain View, CA, USA) as previously described [23].

### Detection of apoptosis by annexin-V staining

The cells were grown for 3 days and treated or not with 10 nM or 100 nM of paclitaxel. Apoptotic cells were detected using the Annexin-V-FLUOS Staining Kit (Roche). FITC fluorescence was then analyzed in 2×10^4 ^cells by a FACscan flow cytometer. The percentage of apoptotic cells was determined by analysis with cellQuest^(tm) ^software (Becton Dickinson).

### Caspase 3 activity assay

Briefly, cells were treated or not with 100 nM of paclitaxel for 10 h. Caspase 3 activity was determined using the Caspase-3/CPP32 fluorometric assay kit (Clinisciences, Montrouge, France).

## Competing interests

The authors declare that they have no competing interests.

## Authors' contributions

AT and JAV carried out the cell and molecular biology experiments and drafted the manuscript. LP performed the ABCB1 expression and activity experiments. SEG and SBL carried out the mouse xenograft experiments. EG performed cytotoxicity assays. CC developed the pEGFP-N1-ZNF217 plasmid and the ZNF217 antibody used in this study. MV and AT performed the apoptosis experiments. PAC conceived the study, its design and coordination and drafted the manuscript. All authors critically read the manuscript and approved the final version.

## References

[B1] Quinlan KG Verger A Yaswen P Crossley M Amplification of zinc finger gene 217 (ZNF217) and cancer: when good fingers go bad Biochim Biophys Acta 2007 1775 333 340 17572303 10.1016/j.bbcan.2007.05.001

[B2] Sen S Zhou H White RA A putative serine/threonine kinase encoding gene BTAK on chromosome 20q13 is amplified and overexpressed in human breast cancer cell lines Oncogene 1997 14 2195 2200 10.1038/sj.onc.1201065 9174055

[B3] Lee JM The role of protein elongation factor eEF1A2 in ovarian cancer Reprod Biol Endocrinol 2003 1 69 10.1186/1477-7827-1-69 14588074 PMC239897

[B4] Collins C Rommens JM Kowbel D Godfrey T Tanner M Hwang SI Polikoff D Nonet G Cochran J Myambo K Positional cloning of ZNF217 and NABC1: genes amplified at 20q13.2 and overexpressed in breast carcinoma Proc Natl Acad Sci USA 1998 95 8703 8708 10.1073/pnas.95.15.8703 9671742 PMC21140

[B5] Ginestier C Cervera N Finetti P Esteyries S Esterni B Adelaide J Xerri L Viens P Jacquemier J Charafe-Jauffret E Prognosis and gene expression profiling of 20q13-amplified breast cancers Clin Cancer Res 2006 12 4533 4544 10.1158/1078-0432.CCR-05-2339 16899599

[B6] Ginzinger DG Godfrey TE Nigro J Moore DH Suzuki S Pallavicini MG Gray JW Jensen RH Measurement of DNA copy number at microsatellite loci using quantitative PCR analysis Cancer Res 2000 60 5405 5409 11034080

[B7] Nonet GH Stampfer MR Chin K Gray JW Collins CC Yaswen P The ZNF217 gene amplified in breast cancers promotes immortalization of human mammary epithelial cells Cancer Res 2001 61 1250 1254 11245413

[B8] Chin K de Solorzano CO Knowles D Jones A Chou W Rodriguez EG Kuo WL Ljung BM Chew K Myambo K In situ analyses of genome instability in breast cancer Nat Genet 2004 36 984 988 10.1038/ng1409 15300252

[B9] Li P Maines-Bandiera S Kuo WL Guan Y Sun Y Hills M Huang G Collins CC Leung PC Gray JW Multiple roles of the candidate oncogene ZNF217 in ovarian epithelial neoplastic progression Int J Cancer 2007 120 1863 1873 10.1002/ijc.22300 17266044

[B10] Collins C Volik S Kowbel D Ginzinger D Ylstra B Cloutier T Hawkins T Predki P Martin C Wernick M Comprehensive genome sequence analysis of a breast cancer amplicon Genome Res 2001 11 1034 1042 10.1101/gr.GR1743R 11381030 PMC311107

[B11] Banck MS Li S Nishio H Wang C Beutler AS Walsh MJ The ZNF217 oncogene is a candidate organizer of repressive histone modifiers Epigenetics 2009 4 100 106 10.4161/epi.4.2.7953 19242095 PMC2929765

[B12] Cowger JJ Zhao Q Isovic M Torchia J Biochemical characterization of the zinc-finger protein 217 transcriptional repressor complex: identification of a ZNF217 consensus recognition sequence Oncogene 2007 26 3378 3386 10.1038/sj.onc.1210126 17130829

[B13] Quinlan KG Nardini M Verger A Francescato P Yaswen P Corda D Bolognesi M Crossley M Specific recognition of ZNF217 and other zinc finger proteins at a surface groove of C-terminal binding proteins Mol Cell Biol 2006 26 8159 8172 10.1128/MCB.00680-06 16940172 PMC1636751

[B14] Huang G Krig S Kowbel D Xu H Hyun B Volik S Feuerstein B Mills GB Stokoe D Yaswen P ZNF217 suppresses cell death associated with chemotherapy and telomere dysfunction Hum Mol Genet 2005 14 3219 3225 10.1093/hmg/ddi352 16203743

[B15] Sun G Zhou J Yin A Ding Y Zhong M Silencing of ZNF217 gene influences the biological behavior of a human ovarian cancer cell line Int J Oncol 2008 32 1065 1071 18425333

[B16] Sun Y Wong N Guan Y Salamanca CM Cheng JC Lee JM Gray JW Auersperg N The eukaryotic translation elongation factor eEF1A2 induces neoplastic properties and mediates tumorigenic effects of ZNF217 in precursor cells of human ovarian carcinomas Int J Cancer 2008 123 1761 1769 10.1002/ijc.23708 18661515 PMC2606039

[B17] McGrogan BT Gilmartin B Carney DN McCann A Taxanes, microtubules and chemoresistant breast cancer Biochim Biophys Acta 2008 1785 96 132 18068131 10.1016/j.bbcan.2007.10.004

[B18] Mackay A Tamber N Fenwick K Iravani M Grigoriadis A Dexter T Lord CJ Reis-Filho JS Ashworth A A high-resolution integrated analysis of genetic and expression profiles of breast cancer cell lines Breast Cancer Res Treat 2009 118 481 498 10.1007/s10549-008-0296-7 19169812

[B19] Shadeo A Lam WL Comprehensive copy number profiles of breast cancer cell model genomes Breast Cancer Res 2006 8 R9 10.1186/bcr1370 16417655 PMC1413994

[B20] Jang SH Wientjes MG Au JL Kinetics of P-glycoprotein-mediated efflux of paclitaxel J Pharmacol Exp Ther 2001 298 1236 1242 11504826

[B21] Martin C Berridge G Higgins CF Mistry P Charlton P Callaghan R Communication between multiple drug binding sites on P-glycoprotein Mol Pharmacol 2000 58 624 632 10953057 10.1124/mol.58.3.624

[B22] Shapiro AB Ling V Positively cooperative sites for drug transport by P-glycoprotein with distinct drug specificities Eur J Biochem 1997 250 130 137 10.1111/j.1432-1033.1997.00130.x 9432000

[B23] Wang EJ Casciano CN Clement RP Johnson WW In vitro flow cytometry method to quantitatively assess inhibitors of P-glycoprotein Drug Metab Dispos 2000 28 522 528 10772630

[B24] Wang EJ Casciano CN Clement RP Johnson WW Active transport of fluorescent P-glycoprotein substrates: evaluation as markers and interaction with inhibitors Biochem Biophys Res Commun 2001 289 580 585 10.1006/bbrc.2001.6000 11716514

[B25] Jordan MA Mechanism of action of antitumor drugs that interact with microtubules and tubulin Curr Med Chem Anticancer Agents 2002 2 1 17 10.2174/1568011023354290 12678749

[B26] Jordan MA Wendell K Gardiner S Derry WB Copp H Wilson L Mitotic block induced in HeLa cells by low concentrations of paclitaxel (Taxol) results in abnormal mitotic exit and apoptotic cell death Cancer Res 1996 56 816 825 8631019

[B27] Kutuk O Letai A Alteration of the mitochondrial apoptotic pathway is key to acquired paclitaxel resistance and can be reversed by ABT-737 Cancer Res 2008 68 7985 7994 10.1158/0008-5472.CAN-08-1418 18829556 PMC2603173

[B28] Noguchi S Predictive factors for response to docetaxel in human breast cancers Cancer Sci 2006 97 813 820 10.1111/j.1349-7006.2006.00265.x 16805818 PMC11158941

[B29] Anand S Penrhyn-Lowe S Venkitaraman AR AURORA-A amplification overrides the mitotic spindle assembly checkpoint, inducing resistance to Taxol Cancer Cell 2003 3 51 62 10.1016/S1535-6108(02)00235-0 12559175

[B30] Blagosklonny MV How cancer could be cured by 2015 Cell Cycle 2005 4 269 278 10.4161/cc.4.11.2208 15655345

[B31] Hotchkiss RS Strasser A McDunn JE Swanson PE Cell death N Engl J Med 2009 361 1570 1583 10.1056/NEJMra0901217 19828534 PMC3760419

[B32] Ibrado AM Liu L Bhalla K Bcl-xL overexpression inhibits progression of molecular events leading to paclitaxel-induced apoptosis of human acute myeloid leukemia HL-60 cells Cancer Res 1997 57 1109 1115 9067280

[B33] Strobel T Tai YT Korsmeyer S Cannistra SA BAD partly reverses paclitaxel resistance in human ovarian cancer cells Oncogene 1998 17 2419 2427 10.1038/sj.onc.1202180 9824152

[B34] Wang XX Liu R Jin SQ Fan FY Zhan QM Overexpression of Aurora-A kinase promotes tumor cell proliferation and inhibits apoptosis in esophageal squamous cell carcinoma cell line Cell Res 2006 16 356 366 10.1038/sj.cr.7310046 16617331

[B35] Yao JE Yan M Guan Z Pan CB Xia LP Li CX Wang LH Long ZJ Zhao Y Li MW Aurora-A down-regulates IkappaBalpha via Akt activation and interacts with insulin-like growth factor-1 induced phosphatidylinositol 3-kinase pathway for cancer cell survival Mol Cancer 2009 8 95 10.1186/1476-4598-8-95 19891769 PMC2780390

[B36] Scharer CD Laycock N Osunkoya AO Logani S McDonald JF Benigno BB Moreno CS Aurora kinase inhibitors synergize with paclitaxel to induce apoptosis in ovarian cancer cells J Transl Med 2008 6 79 10.1186/1479-5876-6-79 19077237 PMC2614415

[B37] Littlepage LE Wu H Andresson T Deanehan JK Amundadottir LT Ruderman JV Identification of phosphorylated residues that affect the activity of the mitotic kinase Aurora-A Proc Natl Acad Sci USA 2002 99 15440 15445 10.1073/pnas.202606599 12422018 PMC137735

[B38] Castro A Arlot-Bonnemains Y Vigneron S Labbe JC Prigent C Lorca T APC/Fizzy-Related targets Aurora-A kinase for proteolysis EMBO Rep 2002 3 457 462 10.1093/embo-reports/kvf095 11964384 PMC1084108

[B39] Littlepage LE Ruderman JV Identification of a new APC/C recognition domain, the A box, which is required for the Cdh1-dependent destruction of the kinase Aurora-A during mitotic exit Genes Dev 2002 16 2274 2285 10.1101/gad.1007302 12208850 PMC186670

[B40] Fukuda T Mishina Y Walker MP DiAugustine RP Conditional transgenic system for mouse aurora a kinase: degradation by the ubiquitin proteasome pathway controls the level of the transgenic protein Mol Cell Biol 2005 25 5270 5281 10.1128/MCB.25.12.5270-5281.2005 15923640 PMC1140609

[B41] Cha TL Chuang MJ Wu ST Sun GH Chang SY Yu DS Huang SM Huan SK Cheng TC Chen TT Dual degradation of aurora A and B kinases by the histone deacetylase inhibitor LBH589 induces G2-M arrest and apoptosis of renal cancer cells Clin Cancer Res 2009 15 840 850 10.1158/1078-0432.CCR-08-1918 19188154

[B42] Lim SK Gopalan G Aurora-A kinase interacting protein 1 (AURKAIP1) promotes Aurora-A degradation through an alternative ubiquitin-independent pathway Biochem J 2007 403 119 127 10.1042/BJ20061272 17125467 PMC1828899

[B43] Vendrell JA Magnino F Danis E Duchesne MJ Pinloche S Pons M Birnbaum D Nguyen C Theillet C Cohen PA Estrogen regulation in human breast cancer cells of new downstream gene targets involved in estrogen metabolism, cell proliferation and cell transformation J Mol Endocrinol 2004 32 397 414 10.1677/jme.0.0320397 15072547

[B44] Vendrell JA Bieche I Desmetz C Badia E Tozlu S Nguyen C Nicolas JC Lidereau R Cohen PA Molecular changes associated with the agonist activity of hydroxy-tamoxifen and the hyper-response to estradiol in hydroxy-tamoxifen-resistant breast cancer cell lines Endocr Relat Cancer 2005 12 75 92 10.1677/erc.1.00899 15788640

